# Clonal hematopoiesis in patients with rheumatoid arthritis

**DOI:** 10.1038/s41408-018-0107-2

**Published:** 2018-07-26

**Authors:** Paula Savola, Sofie Lundgren, Mikko A. I. Keränen, Henrikki Almusa, Pekka Ellonen, Marjatta Leirisalo-Repo, Tiina Kelkka, Satu Mustjoki

**Affiliations:** 1 Hematology Research Unit Helsinki, University of Helsinki and Department of Hematology, Helsinki University Hospital Comprehensive Cancer Center, Helsinki, Finland; 2 Department of Clinical Chemistry and Hematology, University of Helsinki, Helsinki, Finland; 3 Institute for Molecular Medicine Finland (FIMM), HILIFE; University of Helsinki, Helsinki, Finland; 4 Rheumatology, University of Helsinki and Helsinki University Hospital, Helsinki, Finland

Clonal hematopoiesis (CH) is a phenomenon in which somatic mutations originating from hematopoietic progenitors are detected in peripheral blood cells^1,2^. The prevalence of CH increases with age, and CH can be detected in 10–25% of healthy, elderly individuals^1–4^. Very sensitive methods may allow somatic mutation detection in blood cells in 95% of 50–60-year olds^5^.

CH confers risk for myeloid malignancy and death^1,2^. In addition, recent data has suggested that CH is also a risk factor for cardiovascular disease^1,6,7^. In mice, *Tet2* loss-of-function in myeloid cells alone promoted atherosclerosis and proinflammatory cytokine production^6^. Proinflammatory and dysregulated immune responses play roles in the pathogenesis of multiple diseases. As an example, rheumatoid arthritis (RA) is a chronic autoimmune disease which leads to joint destruction. Despite improved disease outcomes with modern treatments, RA patients have increased risk for death and cardiovascular disease. CH has not been studied in the context of RA previously, except for one study containing self-reported disease history of arthritis^3^.

Aplastic anemia (AA) and hypoplastic myelodysplastic syndrome (hMDS) are characterized by hypocellular bone marrow and peripheral-blood cytopenias, and are difficult to distinguish^8^. Although T cells cause hematopoietic stem cell destruction in AA, cytogenetic abnormalities occur in 4–11%, and CH occurs in up to 50% of AA cases^8^. AA confers a substantial risk for hematological malignancy^8^.

In this project, we aimed to characterize CH in patients with RA. Results were also compared with the data from patients with AA and hMDS, immune-mediated diseases which have established links with CH. In addition, we hypothesized that CH may modulate chronic inflammation or disease activity in RA. Thus, we compared mutation findings with clinical parameters to investigate associations between CH and the clinical phenotype in RA.

We collected peripheral blood samples from 59 RA patients who fulfilled the ACR2010 classification criteria for RA and had been monitored in the Helsinki University Hospital rheumatology outpatient clinic after RA diagnosis. Twelve acquired AA and hMDS patients were recruited from the Helsinki University Hospital hematology clinic. We also used samples from two young healthy controls (aged 18–22) as negative controls. All patients gave written informed consent. The ethical board of our institution approved the study and the declaration of Helsinki principles were followed. Due to descriptive nature of our study, no power calculations were performed.

To detect CH, we designed a custom sequencing panel based on Illumina’s TruSeq Custom Amplicon technology (Illumina, San Diego, CA, USA). The panel was designed as 250 base-pair amplicons with Illumine Design Studio, and it comprised of 583 amplicons. The panel covered not only genes that are commonly mutated in CH in healthy individuals but also typically in AA patients. Coding exons of 34 tumor suppressor genes and/or mutational hotspots were sequenced (Supplementary table 1; exact genomic coordinates provided as Supplementary Data). Sequencing was performed with the Illumina HiSeq2500 system with 150 paired-end reads, and the average coverage for each amplicon is shown in the Supplementary figure 1. Peripheral-blood DNA (250 ng) was used for sequencing, but for AA/hMDS patients, bone-marrow mononuclear cells (MNC) were used, except for one patient (MDS1, sample type was peripheral blood).

Sequencing data was analyzed by previously described methods^9^. Briefly, sequencing reads were aligned to the Hg19 genome with Bowtie2 and GATK IndelRealigner, but bases with Phred score < 20 were excluded from further analyses. Variants were required to have sequencing depth > 500, variant base count > 20, and comprise over 80% of all variant bases in the position. Mismapped variants and variants within 5 base-pairs of a 5 base-pair homopolymer were also discarded. Variant with a variant-allele frequency > 35%, population variants with a population frequency of over 1%, and variants that occurred in more than ten individuals were discarded as germline variants. The variants were annotated with the Ensembl Variant Effect Predictor. The Supplementary Material contains more detailed information on variant calling and filtering.

Normal distribution of the data was investigated graphically and with the Shapiro–Wilk test. Statistical tests include two-sided Mann–Whitney test and Fisher’s exact test for comparisons between groups. *P*-values < 0.05 were considered statistically significant. Longitudinal data was analyzed as In-transformed data with a linear mixed model in SPSS using the unstructured covariance type. Sidak correction was used for paired multiple comparisons in the following analyses. Statistical analyses were performed with Graphpad Prism 6 (Graphpad Software, La Jolla, CA, USA) and SPSS Statistics v.23 (IBM, Armonk, NY, USA).

We discovered CH at 17% prevalence in RA and at 33% in AA/hMDS, which are consistent with previous reports^1–4,8^. The identified mutations fulfilled the criteria for clonal hematopoiesis of indeterminate potential (CHIP)^10^. All synonymous and non-coding mutations were discarded along with mutations with < 2% variant allele frequency (VAF), because we defined CH as a process in which mutations provide survival advantage to cells^11^. The 2% VAF cutoff has also been suggested to define CHIP^10^. We did not require missense mutations to occur in cancer gene databases or have deleterious prediction scores by in silico tools, because these strategies will cause underreporting of novel variants and over-reporting of known variants.

In RA patients, *DNMT3A* mutations were the most common, *TET2* mutations ranking second (Figure 1a; Table 1 and Supplementary table 2), consistently with the mutational spectrum found previously in healthy controls^1,3,4^. None of the *DNMT3A* mutations occurred in the R882 AML/MDS hotspot that has previously been described in CH^1,2,7^. Frameshift and nonsense mutations comprised 8/12 of all mutations in RA patients (Fig. 1b), and these disruptive mutations had higher VAFs than missense mutations (Fig. 1c; *p* = 0.0191). This finding suggests that these mutations give survival advantage to the affected clones.

**Fig. 1:**
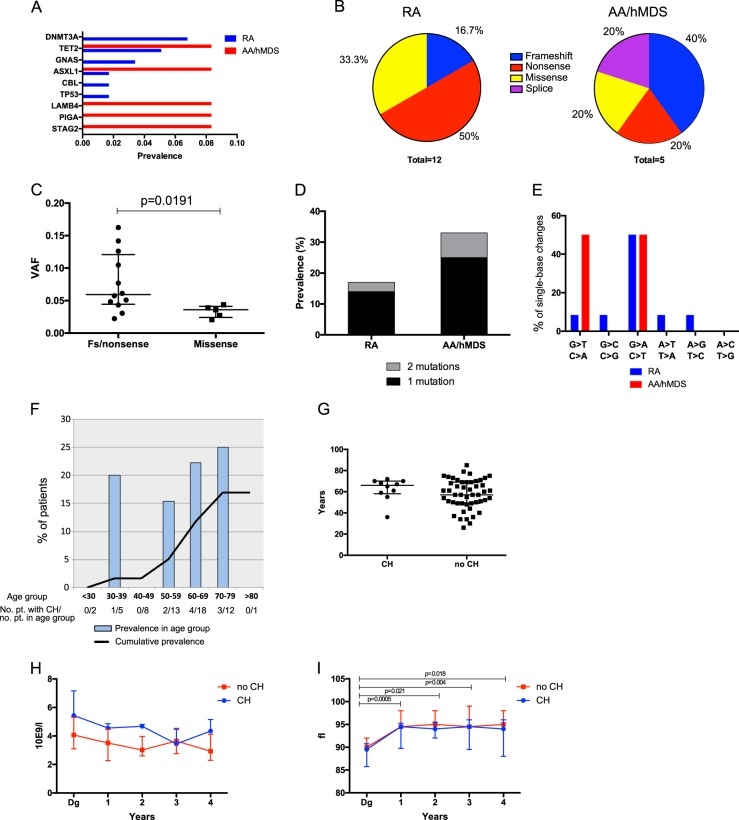
*DNMT3A* and *TET2* mutations are the most common mutations in rheumatoid arthritis patients. **a** The prevalence of patients with mutations in different genes. **b** The percentages of frameshift, nonsense, missense, and splice site mutations identified in RA and AA/hMDS patients. **c** Truncating mutations have higher VAFs than missense mutations (Mann–Whitney test *P* = 0.0191). **d** The overall prevalence (reported as percentage) of CH in RA and AA/hMDS are shown, and the proportions of patients with one or multiple mutations are shown in color. **e** The percentage of different single-nucleotide base changes of all single-base changes. **f** The cumulative prevalence of CH (as percentage) in RA patients. The bars show the percentage of patients with CH in different age groups. The absolute numbers of patients with CH and the number of patients in each age group are also shown in the figure. **g** The median ages of RA patients with/without CH did not show statistically significant difference (Mann–Whitney test *P* = 0.31). **h** The neutrophil counts of RA patients seem to decrease during follow-up, but there was no difference between patients with/without CH. **i** The mean corpuscular volume (MCV) increases during follow-up in RA patients, but there was no difference between patient with/without CH. The analysis was performed using a linear mixed model and the *P*-values are based on post-hoc tests (Sidak correction) on time as a main effect. CH, clonal hematopoiesis; VAF, variant allele frequency; fs, frameshift

**Table 1:** Clonal hematopoiesis mutations discovered in the study

Pt. ID	Age at sampling	Gene	Mutation	AA change	VAF	SIFT/Polyphen	Smoking history	Cancer	Medication before sampling	Max MTX dose (mg/week)
RA1	70	GNAS	20:g.57428923G>A	S138N	0.021	0.19/0.27	Ex-smoker	No	MTX, OXI, iaCS	25
RA2	72	TET2	4:g.106155319_106155320insT	V74fs	0.048	NA	Never	No	MTX, OXI, iaCS	25
RA2	36	CBL	11:g.119149250C>T	R420X	0.022	NA	Smoker	No	MTX, OXI, iaCS	25
RA3	68	GNAS	20:g.57429086G>A	A256T	0.027	0.16/0.001	Ex-smoker	No	MTX, OXI, SA, CS, iaCS, golimumab, etanercept	25
RA4	61	TET2	4:g.106157845C>T	Q916X	0.10	NA	Ex-smoker	No	MTX, OXI	10
RA5	67	DNMT3A	2:g.25459850_25459856delATCATTC	V809fs	0.031	NA	Smoker	No	MTX, OXI, SA, CS	25
RA6	55	TP53	17:g.7574003G>A	R342X	0.0432	NA	Smoker	Yes^a^	MTX, OXI, SA, CS	25
RA7	65	DNMT3A	2:g.25467117G>T	C586X	0.061	NA	Ex-smoker	No	MTX, OXI, CS	25
RA8	70	TET2	4:g.106197060C>G	S1798X	0.057	NA	Smoker	No	MTX, iaCS, abatacept	20
RA9	59	DNMT3A	2:g.25470590A>G	L295P	0.036	0/1	Never	No	MTX, OXI	20
RA9	70	ASXL1	20:g.31022367A>T	K618X	0.077	NA	Ex-smoker	No	MTX, OXI	20
RA10	72	DNMT3A	2:g.25462038C>T	R790K	0.044	0/0.88	Never	No	none	NA
AA1	53	LAMB4	7:g.107688489C>T	R1397Q	0.021	0.63/0	Ex-smoker	No	CS	NA
AA1	53	LAMB4	7:g.107688489C>T	R1397Q	0.039	0.63/0	Ex-smoker	No	CS + CyA	NA
AA2	54	PIGA	X:g.15342786_15342786delC	Splice; impact high	0.13	NA	Never	No	none	NA
AA4	64	ASXL1	20:g.31022288C>A	Y591X	0.14	NA	Smoker	No	none	NA
AA4	64	TET2	4:g.106157409_106157413delAAGAG	Q770fs	0.16	NA	Smoker	No	none	NA
MDS4	57	STAG2	X:g.123202468_123202468delC	H774fs	0.051	NA	Ex-smoker	No	none	NA

Table 1 shows information on patients with clonal hematopoiesis. Additional patient information is presented in the Supplementary material. The mutations are shown in HGVS format in the Hg19 reference genome coordinate system. The amino acid change is shown for the canonical transcript (the CCDS transcript with the longest translation) or for the transcript in which the mutation has the highest impact. Transcript IDs and more detailed information are shown in Supplementary table 2. SIFT and Polyphen scores are prediction scores for missense mutations on the effect for the protein. Both are scaled from 0 to 1. For SIFT, 0 corresponds to deleterious and 1 tolerated. For Polyphen, 1 corresponds to damaging and 0 benign. Disease-modifying medication history before the samples were obtained is included in the table. AA, amino acid; VAF, variant allele frequency; NA, not applicable; Max, maximum; MTX, methotrexate; OXI, hydroxychloroquine; SA, sulfasalazine; CS, corticosteroids; iaCS, intra-articular corticosteroids; CyA, cyclosporine A

^a^Cervical cancer treated with surgery. The patient did not receive chemotherapy, or radiotherapy

AA/hMDS patients (Supplementary tables 3–4) were characterized by mutations in genes such as *LAMB4*, *PIGA*, and *STAG2*, which differentiates them from RA patients (Fig. 1a) and healthy controls^8^. Consistently with RA patients, most AA patients harbored only one CH mutation per patient, but one AA patient harbored two CH mutations (Fig. 1d). Follow-up samples were available from two AA/hMDS patients (AA1 and AA3). One of these patients (AA1) harbored a *LAMB4* mutation that occurred in one follow-up sample but disappeared after anti-thymocyte globulin treatment (Supplementary figure 2).

Extremely low VAFs are challenging to detect even with modern sequencing methods. High sequencing coverage is not the only solution to achieve sensitive mutation detection, because sequencing library preparation can induce mutational artifacts, especially C>A transversions, to the library DNA^12^. With a VAF cutoff at 2%, half of the single-nucleotide changes were C>T mutations (Fig. 1e) in our RA data, supporting the accuracy of mutation calling^1–3,11^.

The prevalence of CH increased with age in RA patients: the overall prevalence of CH was 17% but in 70–79-year olds it increased up to 25% (Fig. 1f). However, patients with CH were not significantly older at sampling (Fig. 1g). Nearly all studied RA patients were treated with anti-rheumatic drugs before sample collection. The patients’ treatment histories included methotrexate in 98%, hydroxychloroquine in 75%, sulfasalazine in 58%, leflunomide in 10%, and biological drugs in 8.5% of cases. No differences in the treatment histories were observed between patients with or without CH (Table 1). Taken together, the signature of CH in RA reflects the ageing hematopoietic system with C>T transitions and mutations in epigenetic regulators^11^.

As chronic inflammation may cause mutagenesis via DNA damage^13^, we also aimed to explore if CH is linked with disease severity. In our cohort (*n* = 59) we could not detect any differences in clinical parameters (such as smoking status, serostatus, disease activity at diagnosis or other autoimmune disease) between RA patients with/without CH (Supplementary figures 3–4; Supplementary tables 5–6). Similarly, during four years of follow-up, no differences emerged in blood cell indices between RA patients with/without CH (neutrophil counts and mean corpuscular volume (MCV) are shown in Fig. 1h, i; Supplementary figure 4; Supplementary tables 7–10). This is in line with previous findings in healthy controls, as CH does not associate with cytopenias in hematologically unselected patients^1^. However, it should be noted that our dataset may lack statistical power to discover subtle changes.

CH mutations occur mostly in myeloid cells, but mutations in lymphoid cells may also modulate autoimmune responses. We recently discovered CD8+ T cells harboring somatic mutations in immune-related genes in RA patients^14^. Similarly, AA- and Felty’s syndrome (RA with neutropenia and splenomegaly) patients’ CD8+ T cells harbor somatic mutations^9,15^. Future research is needed to address the “chicken or the egg” dilemma: does autoimmunity increase mutation formation or do mutations promote inflammation and autoimmunity?

Taken together, CH with a typical mutation profile occurs in RA, but despite of years long systemic inflammation, the rate of CH is not markedly increased. To our knowledge, this is the first report describing the occurrence of CH in RA; one previous study included patients with self-reported disease history but did not find a significant association with self-reported arthritis and CH^3^. However, this data is preliminary, and conclusive results would require analysis of a larger cohort of patients. In addition, although no associations existed between CH and clinical severity of RA in our data, future studies should assess if a specific mutation or a subset of mutations could impact autoimmunity or treatment responses. This is plausible, as CH shapes immune responses in other disease contexts: it increases the risk for cardiovascular endpoints^1,7^, and *Tet2* deficiency in myeloid cells promotes atherosclerosis and proinflammatory cytokine production in mice^6^. Thus, understanding the functional consequences of all CH-associated mutations, and their roles in various disease conditions, is warranted in the future.

## Availability of data and materials

Due to constraints in the ethical permit, the raw sequencing data of patients is only available from the corresponding author upon reasonable request.

## Electronic supplementary material

Supplementary Material

Supplementary Data- Genomic coordinates
