# Author Correction: Clonal hematopoiesis in patients with rheumatoid arthritis

**DOI:** 10.1038/s41408-021-00427-1

**Published:** 2021-02-17

**Authors:** Paula Savola, Sofie Lundgren, Mikko A. I. Keränen, Henrikki Almusa, Pekka Ellonen, Marjatta Leirisalo-Repo, Tiina Kelkka, Satu Mustjoki

**Affiliations:** 1 Hematology Research Unit Helsinki, University of Helsinki and Department of Hematology, Helsinki University Hospital Comprehensive Cancer Center, Helsinki, Finland; 2 Department of Clinical Chemistry and Hematology, University of Helsinki, Helsinki, Finland; 3 Institute for Molecular Medicine Finland (FIMM), HILIFE, University of Helsinki, Helsinki, Finland; 4 Rheumatology, University of Helsinki and Helsinki University Hospital, Helsinki, Finland

**Keywords:** Rheumatic diseases, Haematopoietic stem cells, Cancer genomics, Haematological diseases

Correction to: *Blood Cancer Journal*

10.1038/s41408-018-0107-2 published online 26 July 2018

We detected unintentional small errors in three columns in Table 1 of the published version of the article, which were due to copy-paste errors in the final phase of preparing the table for the submitted manuscript. Errors do not anyhow change the manuscript text or message; only patient age and smoking status were incorrect for some patients.

**Table 1:** Clonal hematopoiesis mutations discovered in the study.

Pt. ID	Age at sampling	Gene	Mutation	AA change	VAF	SIFT/Polyphen	Smoking history	Cancer	Medication before sampling	Max MTX dose (mg/week)
RA1	70	GNAS	20:g.57428923G>A	S138N	0.021	0.19/0.27	ex-smoker	no	MTX, OXI, iaCS	25
RA2	72	TET2	4:g.106155319_106155320insT	V74fs	0.048	NA	never	no	MTX, OXI, iaCS	25
RA2	72	CBL	11:g.119149250C>T	R420X	0.022	NA	never	no	MTX, OXI, iaCS	25
RA3	36	GNAS	20:g.57429086G>A	A256T	0.027	0.16/0.001	smoker	no	MTX, OXI, SA, CS, iaCS, golimumab, etanercept	25
RA4	68	TET2	4:g.106157845C>T	Q916X	0.10	NA	ex-smoker	no	MTX, OXI	10
RA5	61	DNMT3A	2:g.25459850_25459856delATCATTC	V809fs	0.031	NA	ex-smoker	no	MTX, OXI, SA, CS	25
RA6	67	TP53	17:g.7574003G>A	R342X	0.0432	NA	smoker	no	MTX, OXI, SA, CS	25
RA7	55	DNMT3A	2:g.25467117G>T	C586X	0.061	NA	smoker	yes*	MTX, OXI, CS	25
RA8	65	TET2	4:g.106197060C>G	S1798X	0.057	NA	ex-smoker	no	MTX, iaCS, abatacept	20
RA9	70	DNMT3A	2:g.25470590A>G	L295P	0.036	0/1	smoker	no	MTX, OXI	20
RA9	70	ASXL1	20:g.31022367A>T	K618X	0.077	NA	smoker	no	MTX, OXI	20
RA10	59	DNMT3A	2:g.25462038C>T	R790K	0.044	0/0.88	never	no	none	NA
AA1	53	LAMB4	7:g.107688489C>T	R1397Q	0.021	0.63/0	Ex-smoker	no	CS	NA
AA1	53	LAMB4	7:g.107688489C>T	R1397Q	0.039	0.63/0	Ex-smoker	no	CS+CyA	NA
AA2	54	PIGA	X:g.15342786_15342786delC	splice; impact high	0.13	NA	Never	no	none	NA
AA4	64	ASXL1	20:g.31022288C>A	Y591X	0.14	NA	Smoker	no	none	NA
AA4	64	TET2	4:g.106157409_106157413delAAGAG	Q770fs	0.16	NA	Smoker	no	none	NA
MDS4	57	STAG2	X:g.123202468_123202468delC	H774fs	0.051	NA	Ex-smoker	no	none	NA

We apologize for the errors and for the inconvenience this causes, and we ask for a corrigendum for Table 1. We also reviewed the other figures and tables of the manuscript, and these do not include erroneous information.

