# Supplementary material for: Clonal hematopoiesis in patients with rheumatoid arthritis
Source: Blood Cancer J. 2018 Jul 26;8(8):69. doi: 10.1038/s41408-018-0107-2 (PMC6066480; doi:10.1038/s41408-018-0107-2)
Supplement: Supplementary file 1 — Supplementary Material [file 41408_2018_107_MOESM1_ESM.pdf]

Supplementary information

Clonal hematopoiesis in patients with rheumatoid arthritis

Paula Savola, Sofie Lundgren, Mikko A.I. Keränen, Henrikki Almusa, Pekka Ellonen, Marjatta Leirisalo-Repo, Tiina Kelkka, and Satu Mustjoki

Supplementary methods

We obtained peripheral blood samples from 59 rheumatoid arthritis (RA) patients who fulfilled the ACR2010 classification criteria for RA and had been followed up in the Helsinki University Hospital rheumatology outpatient clinic after RA diagnosis. Twelve patients suffering from acquired aplastic anemia (AA) or hypoplastic myelodysplastic syndrome (hMDS) were recruited from the Helsinki University Hospital hematology clinic. We also used two female healthy controls (ages 18-22) as negative controls. All patients gave written informed consent and the declaration of Helsinki principles were followed. The ethical board of our institution approved the study. DNA was extracted from peripheral EDTA blood using magnetic bead-based method by Chemagic MSM1 according to manufacturer's protocol (PerkinElmer, Waltham, MA, USA). For AA/hMDS patients, we used bone-marrow mononuclear cells (MNC), except for one patient (MDS1), from whom we used peripheral whole-blood DNA.

To screen for clonal hematopoiesis (CH), we designed a custom sequencing panel based on the Illumina TruSeq Custom Amplicon technology (Illumina, San Diego, CA, USA). The panel was designed to cover the genes that are commonly mutated in healthy individuals and AA patients[1–8]. Coding exons of 34 tumor suppressor genes and/or mutational hotspots were sequenced (Supplementary table 1; exact genomic coordinates provided as Supplementary Data). 250 base-pair amplicons were designed with Illumina Design Studio. The panel consisted of 583 amplicons.

Library preparation was performed from 250ng genomic DNA according to the manufacturer's instructions (Illumina TruSeq custom Amplicon v1.5 reference guide) with the exception that that the last normalization step was left out to allow exact quantification of the libraries. The sequencing libraries were quantified with Bioanalyzer DNA1000 –chip (Agilent Technologies, Santa Clara, CA, USA).

Sequencing was performed with the Illumina HiSeq2500 system with 150 paired-end (PE) reads (Rapid PE150). The average coverage for each amplicon is shown in Supplementary figure 1. After sequencing, the data was processed by an in-house bioinformatics pipeline, which has been described previously.[9] Bowtie 2 and GATK Indelrealigner were used for sequence alignment to the Hg19 reference genome. Bases with Phred score <20 were discarded. Variants with over 5 supporting reads, less than 2% noise, and positions passing a binomial test threshold p-value $< 10^{-5}$ were called.

- The ratio of the number of variant calls/number of all bases and quality sum/quality sum of all bases at the position was required to be at least 0.9.
- Variants that were called from only one strand (forward or reverse) even though sequencing was performed in that genomic area as paired-end sequencing were discarded.
- Variants with variant-allele frequency (VAF) >35% were discarded as germline variants. We also discarded all variants with a population

frequency of over 1% (based on 1000 Genomes data),[10] and variants that occurred in more than 10 individuals in our dataset.

- Variants with read count<500, variant base count <20, along with variants which comprised less than 80% of all variant bases were discarded.
- Variants within 5 base pairs of a 5 base-pair homopolymer were discarded, as well as clearly mismapped variants (visually inspected in IGV).

The Ensembl GRch37 variant effect predictor[11] was used to annotate variants, including SIFT and Polyphen scores.

In this study, we consider clonal hematopoiesis as a process in which mutations provide cells some survival advantage[12]. Thus, we discarded all non-coding mutations and synonymous mutations from further analyses and required that the VAF must be at least 2%, as this has been suggested as a cutoff threshold for clonal hemaotpoeisis of indeterminate potential (CHIP)[13]. We did not require missense mutations to exist in cancer-gene databases or to harbor deleterious prediction scores in *in silico* analyses, because these strategies will cause underreporting of novel variants and over-reporting of known variants.

Statistical analyses were performed with Graphpad Prism 6 (Graphpad Software, La Jolla, CA, USA) and SPSS Statistics v.23 (IBM, Armonk, NY, USA). Normal distribution of the data was investigated graphically and with the Shapiro-Wilk test. Statistical tests include Mann-Whitney test and Fisher's exact test for comparisons between groups. P-values <0.05 were considered statistically significant. Longitudinal data was analyzed as ln-transformed data with a linear mixed model in SPSS using the unstructured covariance type. Sidak correction was used for paired multiple comparisons in the following analyses.

Supplementary figures and tables

Supplementary table 1. Genes covered by the sequencing panel.

Gene name	Sequenced exons corresponding to canonical	Coding exons in canonical transcript	Comments
DNMT3A	2-23	2-23	
ASXL1	1-13	1-13	
PPM1D	1-2, 4-6	1-6	
SRSF2	1-2	1-2	
TET2	3-11	3-11	
TP53	2-11	2-11	
BCOR	2-15	2-15	
BCORL1	1-12	1-12	
RUNX1	1-8	1-8	
PIGA	1-6	2-6	
EZH2	2-20	2-20	
ETV6	1-8	1-8	
PHF6	2-10	2-10	
ZRSR2	1-11	1-11	
STAG2	3-5, 7-35	3-35	
GATA2	2-6	2-6	
NPM1	1-11	1-11	
CSMD1	1-70	1-70	Gap 42 bp chr8:4495043-4495085<br>Gap 1 bp chr8:3087757-3087758
LAMB4	2-34	2-34	Gap 9 bp chr7:107688552-107688561<br>Gap 54 bp chr7:107748215-107748269<br>Gap 32 bp chr7:107746449-107746481
WT1	1-5, 7-10	1-10	Gap 181 bp chr11:32456412-32456593
CEBPA	1	1	Gap 872 bp chr19: 33792448-33793320
JAK2	12 and 14	3-25	
SF3B1	8-16	1-25	
CBL	8-9, 11	1-16	Gap 24bp chr11:119156251-119156276<br>Gap 3 bp chr11:119156064-19156066
GNAS	1	1-13	Covers 722 bp chr20: 57429568-57428846
IDH2	4 -5	1-11	
IDH1	4-5	3-10	
NRAS	2-3	2-5	
KRAS	2-3	2-5	
U2AF1	2, 5-6, 8	1-8	Gap 12bp chr21:44513223-44513211
SETBP1	4	2-6	Covers 611bp chr 18: 42531686-42532297
MPL	1-12	1-12	
STAT3	12-13, 21	2-24	
STAT5B	16	2-19	

The table shows genes and exons included in the custom sequencing panel. Coding exons are determined based on canonical transcripts (defined according to Ensembl: the transcript with the longest CCDS translation with no stop codons). A detailed list of all genomic positions covered by the panel is provided in Supplementary data.

Supplementary table 2. Details for the mutations detected in this study.

Pt.ID	Ensembl gene	Gene	Mutation	AA change	Transcript	COSMIC	SIFT	PolyPhen	VAF	Coverage
RA1	ENSG00000087460	GNAS	20:g.57428923G>A	S138N	ENST00000306120	-	tolerated_low_confidence(0.19)	benign(0.27)	0.021	1009
RA2	ENSG00000168769	TET2	4:g.106155319_106155320insT	V74fs	ENST00000540549	-	-	-	0.048	33431
RA2	ENSG00000110395	CBL	11:g.119149250C>T	R420X	ENST00000264033	COSM219132	-	-	0.022	13589
RA3	ENSG00000087460	GNAS	20:g.57429086G>A	A256T	ENST00000371100	-	tolerated_low_confidence(0.16)	benign(0.001)	0.027	696
RA4	ENSG00000168769	TET2	4:g.106157845C>T	Q916X	ENST00000540549	COSM43417	-	-	0.105	15408
RA5	ENSG00000119772	DNMT3A	2:g.25459850_25459856delATCATTC	V809fs	ENST00000264709	-	-	-	0.031	4215
RA6	ENSG00000141510	TP53	17:g.7574003G>A	R342X	ENST00000269305	COSM11073	-	-	0.043	509
RA7	ENSG00000119772	DNMT3A	2:g.25467117G>T	C586X	ENST00000264709	-	-	-	0.061	5003
RA8	ENSG00000168769	TET2	4:g.106197060C>G	S1798X	ENST00000540549	COSM5487395	-	-	0.057	20904
RA9	ENSG00000119772	DNMT3A	2:g.25470590A>G	L295P	ENST00000264709	-	deleterious (0)	probably_damaging(1)	0.036	9871
RA9	ENSG00000171456	ASXL1	20:g.31022367A>T	K618X	ENST00000375687	-	-	-	0.077	6566
RA10	ENSG00000119772	DNMT3A	2:g.25462038C>T	R790K	ENST00000264709	-	deleterious (0)	possibly_damaging(0.881)	0.044	6232
AA1	ENSG00000091128	LAMB4	7:g.107688489C>T	R1397Q	ENST00000388781	-	tolerated(0.63)	benign(0)	0.021	10018
AA1	ENSG00000091128	LAMB4	7:g.107688489C>T	R1397Q	ENST00000388781	-	tolerated(0.63)	benign(0)	0.039	13562
AA2	ENSG00000165195	PIGA	X:g.15342786_15342786delC	splice-site	ENST00000333590	-	-	-	0.126	7793
AA4	ENSG00000171456	ASXL1	20:g.31022288C>A	Y591X	ENST00000375687	COSM1681609	-	-	0.142	3188
AA4	ENSG00000168769	TET2	4:g.106157409_106157413delAAGAG	Q770fs	ENST00000540549	-	-	-	0.162	13373
MDS4	ENSG00000101972	STAG2	X:g.123202468_123202468delC	H774fs	ENST00000218089	-	-	-	0.051	6332

The table shows details for all mutations that were identified in this study. All genomic coordinates and annotations are based on the GRCh37 reference genome. The mutations are shown in HGVS format. The amino-acid change for the mutation (AA change) is shown for the canonical transcript or for the transcript that had the most deleterious prediction for the protein structure. Canonical transcripts were defined as the transcript with the longest CCDS translation. The transcript from which the amino-acid changes were determined are also listed in the table. SIFT and Polyphen are *in silico* prediction methods to determine the impact of missense mutations on protein function, and their prediction scores were obtained via the Variant Effect predictor[11] (GRCh37). Abbreviations: Pt. ID, patients identifier; VAF, variant allele frequency; AA, amino acid.

Supplementary table 3. Clinical information on the AA patients.

Pt. ID	Disease	Sex	Age at dg	age at sampling	PNH	LGL lymphocytosis at sampling (yes / no)	TCRγ	BM cellularity	Cytogenetics	Severity at dg	Smoking
AA1	AA	F	52	53	no	1	yes	0,2	46,XX	Moderate	Ex-smoker
AA2	AA	F	52	54	yes	0	yes	0,05	46,XX	Severe	never
AA3	AA	F	40	40	no	0	yes	0,1	46,XX	Moderate	Never
AA4	AA	F	68	64	no	1	NA	0,1	46,XY	Very severe	Smoker
AA6	AA	F	58	60	no	1	no	0,1	46,XX	Moderate	NA
AA7	AA	F	65	64	yes	0	no	NA	46,XX	Moderate	Never
AA8	AA	M	45	45	NA	NA	no	NA	46,XY	severe	Never
AA5	AA	F	66	66	subclinical	0	yes	0,05	46,XX	severe	Never

Clinical information on the eight acquired aplastic anemia patients included in the study. AA1 had also LGL leukemia in addition to AA, and she had two follow-up samples available for sequencing. AA3 also had follow-up samples available, but she did not harbor any mutations in the samples. None of the AA patients had received chemo- or radiotherapy before diagnosis. Abbreviations: AA, aplastic anemia; PNH, paroxysmal nocturnal hemoglobinuria clone; LGL, large granular lymphocyte; TCRγ, TCR gamma chain rearrangement status; BM, bone marrow; F, female; M, male; NA, not available.

Supplementary table 4. Clinical information on hypopolastic MDS patients.

Pt. ID	Disease	Sex	Age at dg	age at sampling	LGL lymphocytosis at sampling (yes / no)	BM cellularity	Cytogenetics	IPPS-R classification	Blast (%)	Smoking
MDS3	ICUS	M	48	48	NA	NA	46,XY	ND	0	Never
MDS1	AA/hMDS	F	37	37	NA	NA	46,XX	Int	4	NA
MDS2	MDS del(5q)	F	45	47	No	0.35	del (5q) (1)	very low	0	Ex-smoker
MDS4	MDS-U	F	57	57	Yes	0.3	inv(3)/t(3q) /del(3q) (3)	low	3	Ex-smoker

Clinical information on the four hypoplastic MDS (hMDS) patients that were included in the study. Only one of the patients had received previous chemo- or radiotherapy: MDS1 had received an allogeneic stem cell transplant for AA 24 years before sample collection. She currently has hMDS diagnosis. In addition, she is the only patient whose sample was peripheral-blood DNA. Other sequenced samples were derived from bone-marrow mononuclear cells. Abbreviations: AA, aplastic anemia; hMDS, hypopolastic MDS; ICUS, idiopathinc cytopenia of undetermined significance; LGL, large granular lymphocyte; BM, bone marrow; IPSS-R, Revised International Prognostic Scoring System; M, male; F, female; ICUS, idiopathic cytopenia(s) of undetermined significance; MDS-U, unclassifiable MDS; ND, not determined; NA, not available.

Supplementary table 5. Clinical data of the RA patients

	Median	IQR	Mean	95% CI
**Age at RA diagnosis**	56.0	46.1-65.5	54.7	51.2-58.3
**Age at sample collection**	61.0	50.0-69.0	58.4	54.9-62.0
**At diagnosis:**				
ESR	17	8-30	22	17-28
CRP	4	3-12	12	7-18
HAQ	0.50	0.22-1.13	0.75	0.56-0.93
DAS28	4.23	3.28-5.04	4.17	3.80-4.54
tender joints	7.5	2-13.8	8.8	6.8-10.9
swollen joints	7	3.5-11.5	8.6	6.7-10.4
Hb	130	124-139	130	128-133
Leuk	7.10	6.15-8.90	7.67	7.07-8.26
Neut	4.39	3.20-5.84	4.57	4.12-5.02
Lymph	2.00	1.71-2.53	2.18	1.96-2.40
Mono	0.57	0.50-0.74	0.64	0.58-0.71

Abbreviations: IQR, interquartile range; CI, confidence interval; ESR, erythrocyte sedimentation rate; HAQ, health assessment questionnaire; DAS28, disease activity score on 28 joints; Hb, hemoglobin (g/l); Leuk, leukocyte count 10E9/l; Neut, neutrophil count 10E9/l; Lymph, lymphocyte count 10E9/l; Mono, monocyte count 10E9/l.

Supplementary table 6. Sex, serostatus, smoking, atherosclerotic events, and pulmonary disease in RA patients

	Count	Percentage (%)	CH (n)	no CH (n)
**Sex**				
female	49	83	10	10
male	10	17	0	39
**Serostatus**				
positive	52	88	9	43
negative	7	12	1	6
**Smoking**				
Smoker	16	27	4	12
ex-smoker	15	25	4	11
never-smoked	24	41	2	22
ND	4	7	0	4
**Other autoimmune disease**				
yes	15	25	4	11
no	44	75	6	38
**Previous cancer**	7	12	1	6
**High LDL**				
yes	35	59	7	28
no	15	25	1	14
ND	9	15	2	7
**Hypertension**				
yes	21	36	3	18
no	25	42	4	21
ND	13	22	3	10
**Diabetes**				
yes	7	12	1	6
no	46	78	9	37
ND	6	10	0	6
**Atherosclerotic disease**	4	7	1	3
**Astma**	10	17	2	8
**Allergy**	10	17	4	6
**COPD**	2	3.4	0	2

Details on comorbidities were collected retrospectively from patient records. High LDL was defined as LDL >3.0mmol/l. Hypertension was defined as a blood pressure chronically over 140/90 mmHg (information extracted from patient records). Atherosclerotic disease was defined as stroke, peripheral atherosclerotic disease or coronary heart disease. No statistically significant associations were observed between RA patients with/without CH. However, it should be noted that 8/10 of patients with CH were smokers or ex-smokers. Abbreviations: ND, not determined; CH, clonal hematopoiesis.

Supplementary table 7. Descriptive statistics for blood cell indices in RA patients in follow-up.

	Total no. of patients	ND	Mean	Median	Minimum	Maximum	IQR lower	IQR upper	95% CI (+/-)	St. dev
**dg-Leuk**	58	1	7.45	7.1	4.1	16.1	6	8.38	0.58	2.25
**1-year-Leuk**	57	2	6.56	6.3	3	15.6	5	7.2	0.62	2.37
**2-year-Leuk**	54	5	6.54	5.9	3.9	14.3	5.13	7.75	0.51	1.91
**3-year-Leuk**	52	7	6.66	6.25	3.7	15.1	5.33	7.83	0.53	1.94
**4-year-Leuk**	39	20	6.55	6	3.8	14.9	5.25	7.15	0.68	2.16
**dg-Trom**	58	1	289.43	272	177	520	234.5	319	20.85	81.01
**1-year-Trom**	57	2	264.47	249	79	539	220	300	19.45	74.93
**2-year-Trom**	54	5	267.70	256.5	153	515	221.5	305.75	18.51	69.40
**3-year-Trom**	52	7	262.71	254.5	140	513	232.5	294.5	16.86	62.04
**4-year-Trom**	39	20	257.95	246	132	508	218	295.5	22.50	71.68
**dg-Neut**	57	2	4.46	4.07	2.08	8.98	3.1	5.49	0.43	1.66
**1-year-Neut**	56	3	3.70	3.565	1.41	9.05	2.52	4.55	0.43	1.65
**2-year-Neut**	51	8	3.52	3.24	1.86	6.64	2.63	4.29	0.32	1.15
**3-year-Neut**	51	8	3.86	3.57	1.87	9.03	2.87	4.53	0.40	1.45
**4-year-Neut**	35	24	3.48	2.99	1.34	8.19	2.35	4.48	0.47	1.43
**dg-Lymf**	57	2	2.10	1.88	0.98	5.53	1.7	2.35	0.20	0.79
**1-year-Lymf**	56	3	1.90	1.86	0.91	4.95	1.41	2.09	0.20	0.77
**2-year-Lymf**	51	8	1.89	1.84	1.03	4.76	1.48	2.13	0.17	0.63
**3-year-Lymf**	51	8	1.96	1.71	0.92	4.7	1.49	2.29	0.21	0.77
**4-year-Lymf**	35	24	1.89	1.67	1.06	5.39	1.38	1.85	0.30	0.92
**dg-Mono**	57	2	0.63	0.58	0.28	1.62	0.49	0.7	0.06	0.23
**1-year-Mono**	56	3	0.61	0.615	0.22	1.19	0.47	0.72	0.05	0.19
**2-year-Mono**	51	8	0.62	0.57	0.3	1.24	0.46	0.78	0.06	0.22
**3-year-Mono**	51	8	0.64	0.61	0.39	1.07	0.48	0.78	0.05	0.18
**4-year-Mono**	35	24	0.62	0.59	0.31	1.12	0.48	0.73	0.06	0.19
**dg-hb**	58	1	130.52	130.5	106	156	124	137.75	2.77	10.76
**1-year-Hb**	56	3	131.14	130.5	98	165	122	138	3.29	12.56
**2-year-Hb**	54	5	131.06	130	109	157	123.25	139.75	2.95	11.04
**3-year-Hb**	54	5	133.24	134	108	156	128	140.75	2.84	10.65
**4-year-Hb**	38	21	134.58	137.5	108	151	129.25	140.75	3.02	9.51
**dg-Eryt**	58	1	4.37	4.38	3.75	5.08	4.16	4.60	0.08	0.31
**1-year-Eryt**	56	3	4.20	4.18	3.35	4.96	3.96	4.47	0.10	0.37
**2-year-Eryt**	54	5	4.19	4.2	3.32	5.01	3.92	4.48	0.10	0.38
**3-year-Eryt**	54	5	4.26	4.3	3.3	4.92	4.03	4.50	0.10	0.36
**4-year-Eryt**	38	21	4.28	4.38	3.32	4.88	4.03	4.58	0.13	0.40
**dg-MCV**	58	1	90.00	90	81	102	87	92	1.14	4.43
**1-year-MCV**	56	3	94.48	94.5	83	107	90	98	1.40	5.33
**2-year-MCV**	54	5	94.24	94	77	106	92	97.75	1.38	5.19
**3-year-MCV**	54	5	94.37	94.5	76	106	92.25	97.75	1.38	5.16
**4-year-MCV**	38	21	94.63	94.5	86	106	91.25	97.75	1.59	4.99
**dg-RDW**	57	2	13.89	14	12	17	13	14	0.25	0.96
**1-year-RDW**	55	4	14.55	14	12	22	14	15	0.41	1.56
**2-year-RDW**	53	6	14.23	14	12	17	13	15	0.34	1.28
**3-year-RDW**	52	7	14.21	14	12	17	14	15	0.30	1.11
**4-year-RDW**	37	22	14.24	14	12	16	14	15	0.32	0.98

Descriptive statistics for the blood cell indices from RA patients. Abbreviations: ND, not determined; IQR, interquartile range; CI, confidence interval; St.dev, standard deviation; dg, diagnosis; Leuk, leukocyte count (10E9/l); Trom, thrombocyte count (10E9/l); Neut, neutrophil count (10E9/l); Lymf, lymphocyte count (10E9/l); Mono, monocyte count (10E9/l); Hb, hemoglobin (g/l); Eryt, erythrocyte count (10E12/l); MCV, mean corpuscular volume (fl); RDW, red cell distribution width (%).

Supplementary table 8. P-values on interactions between clonal hematopoiesis, smoking, and blood cell counts in follow-up.

	CH	Time	Smoking	Time * CH	Time * CH * Smoking	CH * Smoking
Leukocytes	0.459	0.279	0.09	0.852	0.17	0.685
Thrombocytes	0.946	0.144	0.483	0.637	0.398	0.615
Neutrophils	0.364	0.354	0.138	0.206	0.663	0.516
Lymphocytes	0.96	<u>0.014</u>	0.164	0.107	<u>0.008</u>	0.771
Monocytes	0.312	0.294	0.132	0.523	0.268	0.373
Hemoglobin	0.998	0.526	0.642	0.923	0.825	0.174
MCV	0.648	<u>0.00064</u>	0.841	0.94	0.965	0.541
RDW	0.763	0.261	0.521	0.855	0.744	0.861

Blood cell indices of RA patients were determined at diagnosis and annually (12 months +/- 4 months) for four years. The distribution of data was assessed graphically and with Shapiro-Wilk test. After ln-transformation, a mixed model was used to study if longitudinal differences occurred between patients with/without CH. Fixed factors included time, smoking status (smoker/ex-smoker or never smoked) and CH status (mutation/no mutations). P-values of interactions between these factors are shown above. Most interactions were not statistically significant. However, in follow-up, lymphocyte counts are affected by the combination of CH and smoking. Also, in follow-up, MCV is significantly affected, but MCV is not influenced by clonal hematopoiesis or smoking. Statistically significant values are underlined. Abbreviations: RDW, red cell distribution width; MCV, mean corpuscular volume; CH, clonal hematopoiesis.

Supplementary table 9. P-values for the analysis on the effect of smoking and clonal hematopoiesis on lymphocyte counts in follow-up.

		No. of patients	Time	dg vs 1 year	dg vs 2 years	dg vs 3 years	dg vs 4 years
p-values	CH, smoking	8	0.00028	1.00	1.00	0.54	1.00
	CH, no smoking	2	ND	ND	ND	ND	ND
	no CH, smoking	23	0.044	0.93	1.00	0.99	0.96
	no CH, no smoking	22	0.0039	0.004	0.20	0.87	0.031

In an interaction model, the effect of CH and smoking were significant on lymphocyte counts in follow-up (CH*time*smoking), so patients were grouped according to smoking status and clonal hematopoiesis status. Although the effect of time was significant factor on lymphocyte counts in follow-up in different patient groups, the post-hoc tests were not significant in any group except in no CH, no smoking. Post-hoc tests were corrected for multiple comparisons with the Sidak method. Abbreviations: CH, clonal hematopoiesis; dg, diagnosis; ND, not determined (because n=2).

Supplementary table 10. Mean and confidence interval estimates for the analysis on the effect of smoking and clonal hematopoiesis on lymphocyte counts in follow-up.

		dg	1 year	2 years	3 years	4 years
CH, smoking	mean	2.12	2.03	2.01	1.67	2.11
	lower 95% CI	1.54	1.63	1.71	1.38	1.58
	upper 95% CI	2.91	2.52	2.37	2.01	2.82
CH, no smoking	mean	ND	ND	ND	ND	ND
	lower 95% CI	ND	ND	ND	ND	ND
	upper 95% CI	ND	ND	ND	ND	ND
no CH, smoking	mean	2.13	1.97	2.09	2.28	1.98
	lower 95% CI	1.81	1.64	1.76	1.93	1.60
	upper 95% CI	2.51	2.36	2.49	2.69	2.44
no CH, no smoking	mean	1.84	1.57	1.67	1.71	1.58
	lower 95% CI	1.68	1.40	1.49	1.51	1.44
	upper 95% CI	2.01	1.75	1.86	1.93	1.74

The linear mixed model mean and confidence interval estimates for lymphocyte counts in follow-up time-points for each group are shown as back-transformed values. Post-hoc tests were corrected for multiple comparisons with the Sidak method. Abbreviations: CH, clonal hematopoiesis; CI, confidence interval; ND, not determined.

Supplementary figures.

Supplementary figure 1. Average coverage per each amplicon.

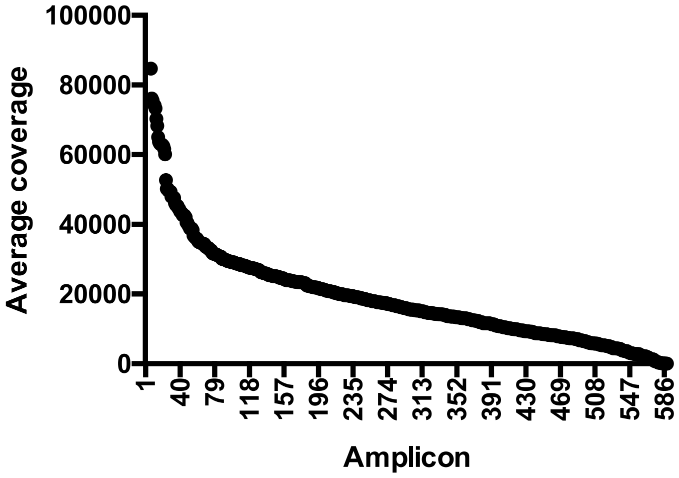

	Coverage
Average	18612
Median	15659
IQR	8699-24677
Range	1,75-84687

The average coverage of each sequenced amplicon is shown. The custom sequencing panel consisted of 583 amplicons, of which 89% reached over 5000x average coverage.

Supplementary figure 2. Mutation VAF and treatment response.

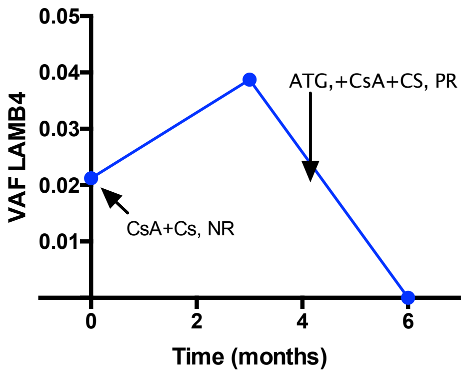

AA1 had received cyclosporine A (CsA) and corticosteroids (CS) as first-line therapy for AA, and she was receiving them when two of the samples were obtained. However, she did not have response (NR) to the CsA+CS treatment. She received antithymocyte globulin (ATG), CsA, and CS some months later. In the sample that was obtained after the ATG treatment, the *LAMB4* mutation could not be detected although it was detected in the previous two samples. She had a partial response (PR) to the ATG+CsA+CS treatment.

Supplementary figure 3. Clinical parameters at diagnosis phase in RA patients with clonal hematopoiesis and in RA patients without clonal hematopoiesis.

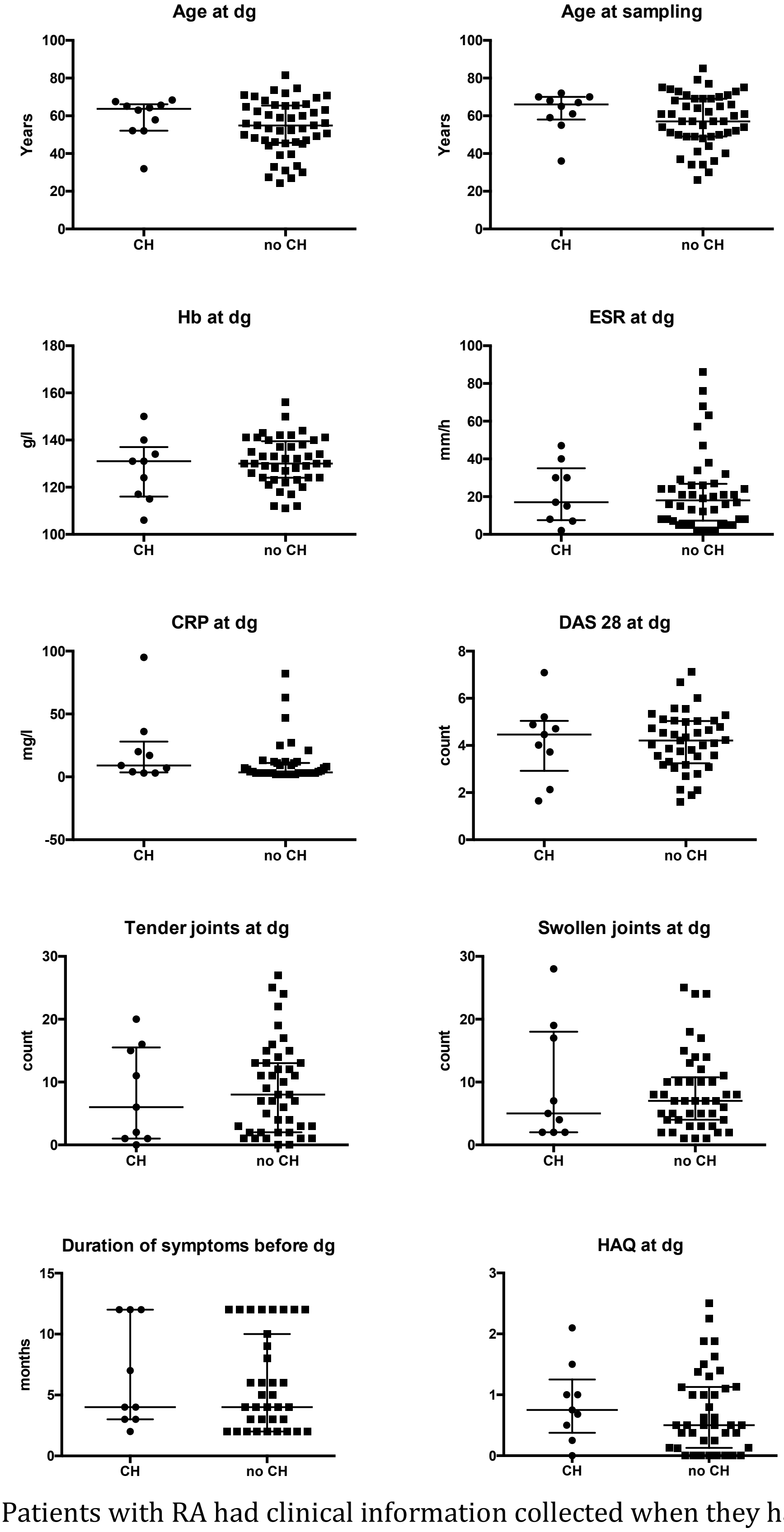

Patients with RA had clinical information collected when they had received RA diagnosis. We collected the samples for sequencing 2-4 years after RA diagnosis,

but we tested the differences between patients with/without CH at diagnosis, because the situations at diagnosis are more comparable than follow-up parameters: after diagnosis, the patients received anti-rheumatic and anti-inflammatory treatments with a goal for disease remission. Also, it is likely that the CH mutations were not restricted to the follow-up samples, but have existed already at diagnosis. Clinical parameters did not differ between patients with clonal hematopoiesis and patients without clonal hematopoiesis (Mann-Whitney test). Abbreviations: CH, clonal hematopoiesis as defined in this study; ESR, erythrocyte sedimentation rate; DAS28, disease activity score in 28 joints; HAQ, health assessment questionnaire.

Supplementary figure 4. Blood cells counts of RA patients in follow-up.

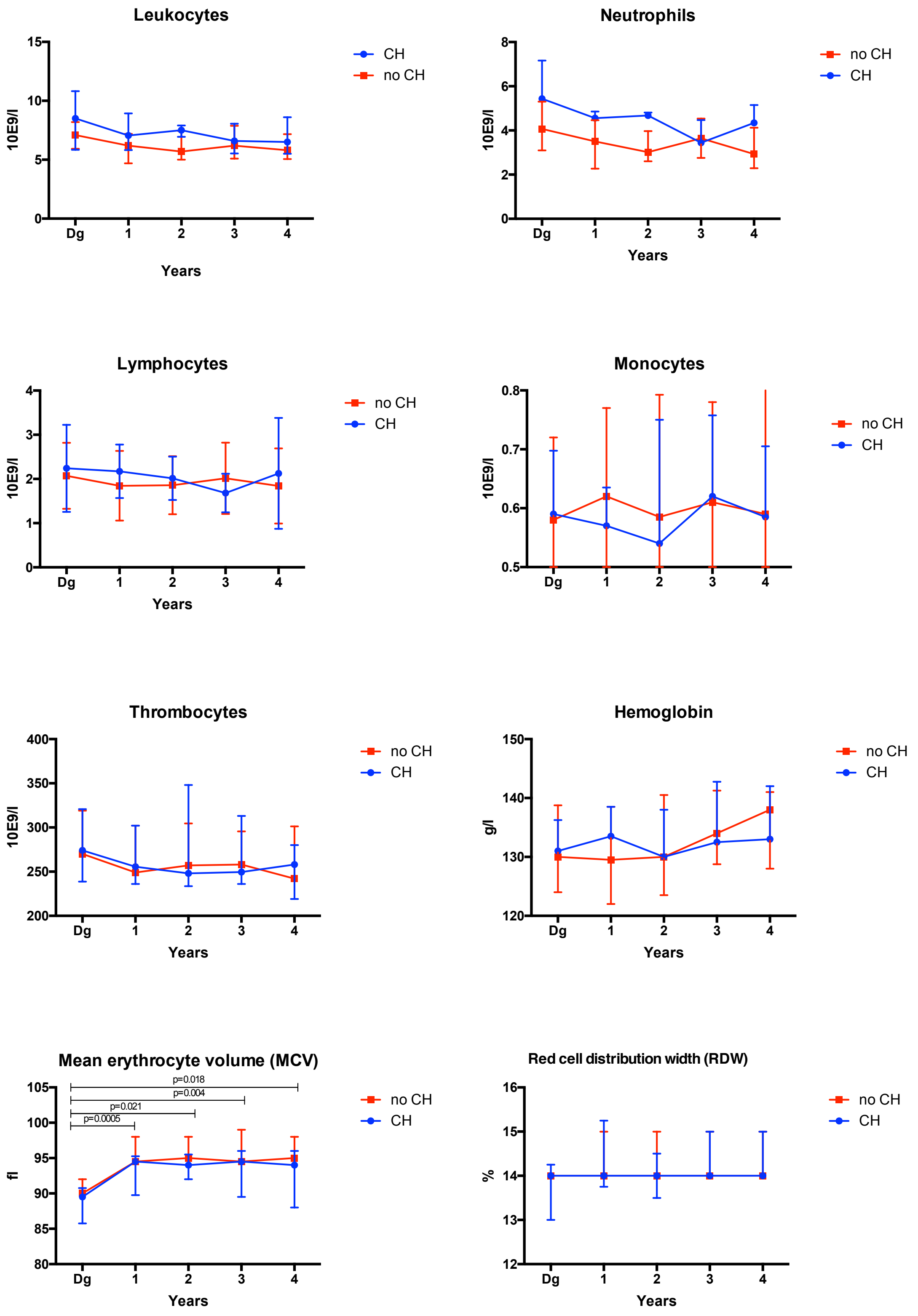

The blood cell counts of all patients were collected from clinical records from diagnosis up to 4 years after RA diagnosis. Blood cell counts or their changes did not differ between patients with clonal hematopoiesis and patients without clonal hematopoiesis. However, there were statistically significant changes in MCV and the lymphocyte count in follow-up (see Supplementary table 7). The

mean MCV at follow-up time-points was significantly elevated from the diagnosis mean (p-values shown in the figure; Sidak correction for multiple comparisons). The plot shows medians and error bars represent the interquartile range. Abbreviations: CH, clonal hematopoiesis as defined in this study; Dg, diagnosis.

Supplementary material references

1 Jaiswal S *et al.* Age-related clonal hematopoiesis associated with adverse outcomes. *N Engl J Med* 2014; **371**: 2488–98.
2 Genovese G *et al.* Clonal Hematopoiesis and Blood-Cancer Risk Inferred from Blood DNA Sequence. *N Engl J Med* 2014; **371**: 2477–2487.
3 McKerrell T *et al.* Leukemia-Associated Somatic Mutations Drive Distinct Patterns of Age-Related Clonal Hemopoiesis. *Cell Rep* 2015; **10**: 1239–1245.
4 Xie M *et al.* Age-related mutations associated with clonal hematopoietic expansion and malignancies. *Nat Med* 2014; **20**: 1472–8.
5 Yoshizato T *et al.* Somatic Mutations and Clonal Hematopoiesis in Aplastic Anemia. *N Engl J Med* 2015; **373**: 35–47.
6 Babushok D V. *et al.* Emergence of clonal hematopoiesis in the majority of patients with acquired aplastic anemia. *Cancer Genet* 2015; **208**: 115–128.
7 Kulasekararaj AG *et al.* Somatic mutations identify a subgroup of aplastic anemia patients who progress to myelodysplastic syndrome. *Blood* 2014; **124**: 2698–2704.
8 Mohamedali AM *et al.* High concordance of genomic and cytogenetic aberrations between peripheral blood and bone marrow in myelodysplastic syndrome (MDS). *Leukemia* 2015; **29**: 1928–1938.
9 Savola P *et al.* Somatic STAT3 mutations in Felty syndrome: an implication for a common pathogenesis with large granular lymphocyte leukemia. *Haematologica* 2018; **103**: 304–312.
10 Auton A *et al.* A global reference for human genetic variation. *Nature* 2015; **526**: 68–74.
11 McLaren W *et al.* The Ensembl Variant Effect Predictor. *Genome Biol* 2016; **17**: 1–14.
12 Bowman RL, Busque L, Levine RL. Clonal Hematopoiesis and Evolution to Hematopoietic Malignancies. *Cell Stem Cell* 2018; **22**: 157–170.
13 Steensma DP *et al.* Clonal hematopoiesis of indeterminate potential and its distinction from myelodysplastic syndromes. *Blood* 2015; **126**: 9–16.
